# Device Modeling and Design of Inverted Solar Cell Based on Comparative Experimental Analysis between Effect of Organic and Inorganic Hole Transport Layer on Morphology and Photo-Physical Property of Perovskite Thin Film

**DOI:** 10.3390/ma14092191

**Published:** 2021-04-24

**Authors:** Xiaolan Wang, Xiaoping Zou, Jialin Zhu, Chunqian Zhang, Jin Cheng, Zixiao Zhou, Haiyan Ren, Yifei Wang, Xiaotong Li, Baokai Ren, Keke Song

**Affiliations:** Beijing Advanced Innovation Center for Materials Genome Engineering, Research Center for Sensor Technology, Beijing Key Laboratory for Sensor, MOE Key Laboratory for Modern Measurement and Control Technology, School of Automation, Beijing Information Science and Technology University, Jianxiangqiao Campus, Beijing 100101, China; wangxl1105@163.com (X.W.); chun-qiancool@163.com (C.Z.); chengjin@bistu.edu.cn (J.C.); 18049217206@163.com (Z.Z.); yanh3100@gmail.com (H.R.); yifewang2020@126.com (Y.W.); xiaotong252240@163.com (X.L.); renbk2021@163.com (B.R.); songmengke163@163.com (K.S.)

**Keywords:** hole transport layer, morphology, photophysical properties, simulation

## Abstract

It is crucial to find a good material as a hole transport layer (HTL) to improve the performance of perovskite solar cells (PSCs), devices with an inverted structure. Polyethylene dioxythiophene-poly (styrene sulfonate) (PEDOT:PSS) and inorganic nickel oxide (NiO_x_) have become hotspots in the study of hole transport materials in PSCs on account of their excellent properties. In our research, NiO_x_ and PEDOT: PSS, two kinds of hole transport materials, were prepared and compared to study the impact of the bottom layer on the light absorption and morphology of perovskite layer. By the way, some experimental parameters are simulated by wx Analysis of Microelectronic and Photonic Structures (wxAMPS). In addition, thin interfacial layers with deep capture levels and high capture cross sections were inserted to simulate the degradation of the interface between light absorption layer and PEDOT:PSS. This work realizes the combination of experiment and simulation. Exploring the mechanism of the influence of functional layer parameters plays a vital part in the performance of devices by establishing the system design. It can be found that the perovskite film growing on NiO_x_ has a stronger light absorption capacity, which makes the best open-circuit voltage of 0.98 V, short-circuit current density of 24.55 mA/cm^2^, and power conversion efficiency of 20.01%.

## 1. Introduction

For the past few years, the enhancement of perovskite solar cells (PSCs) in device performance has benefited from a large number of experimental and theoretical studies, which are aimed at the structure of devices and selection of materials [1,2,3,4,5,6,7,8,9]. In the fabrication of PSCs, the photophysical properties and morphology of the light absorption layer are critical factors affecting the performance of the entire device [10,11,12,13,14,15,16,17,18,19]. The bottom substrate will affect the perovskite film quality regardless of perovskite composition and preparing process [20,21]. Owing to their low hysteresis, inverted PSCs have attracted much attention, in which the selection of hole transport layer (HTL) is even more significant [22,23].

Guo et al. first reported inverted PSCs by employing polyethylene dioxythiophene-poly (styrene sulfonate) (PEDOT:PSS) as the HTL and achieved the power conversion efficiency (PCE) of 3.9% [24]. The perovskite film fabricated by Sun et al. by introducing the two-step method is thicker and denser and the prepared device with the PCE of 7.4% is achieved [25]. However, PEDOT: PSS was acid and hygroscopic in nature, leading to the degradation of perovskite layer, which would seriously affect the performance and stability of PSCs in a humid atmosphere [26,27,28,29]. Hence, other inorganic materials were adopted as HTLs, such as ZnO, CuSCN, NiO_x_, Cu_2_O, etc. [30,31,32,33,34]. PSCs assembled using NIO_x_ as HTL have superior ambient stability [35]. Irwin et al. were the first to use NIO_x_ instead of PEDOT: PSS [32] demonstrated solar cells. In the study of Chen et al., the device with 3% K-doped NiO_x_ as HTL had the optimal property, with a top PCE value of 17.05% and a filling coefficient of 74% [36].Wang et al. increased PCE with the increase of short-circuit current by preparing devices with the inverted structure [37]. Lian et al. further improved the performance of NiO_x_/PMMA-based devices by improving the interaction of methoxy groups with Pb^2+^ and carbonyl and the interface contact [38]. Compared with PEDOT:PSS, NiO_x_ has a higher potential difference between the two kinds of electric charge transport layers because it has a deeper valence band which can generate an improved ohmic contact with the perovskite film.

Meredith et al. have investigated the perovskite structures deposited on different p-type organic transport layers [21]. The investigations display that the growth of perovskite layer has a great relationship with the substrate. The perovskite films grown on PEDOT:PSS might display pinholes and incomplete coverage as demonstrated in previous reports [20,23]. As a vital theoretical support for the research of device performance, numerical simulation helps us to make out the internal mechanism of devices in depth. The simulation designs of PSCs have been performed in many investigations, which modulate the device structure and film parameters for device optimization [1,2,3]. Some studies have indicated that the selection of hole transport materials affects the device performance in simulation design [2,7]. The alignments of the valence bands of perovskite layer and the HTL varies due to the differences in mobilities, band gap energies and electron affinities of these materials [7]. Furthermore, the high defect density of interface layer and the the perovskite film also generate opposite forces on the device’s performance, which is ascribed to the increase in charge carrier recombination [3,6,8,10].

In this study, two kinds of HTL materials were selected to research the impact of bottom layers on optical absorption and morphology of perovskite films. As the mainstream materials of the hole transport layer, they still have great research value. We used the test data from the film we made to set more real parameters in the simulation software, so that the simulation experiment results of the device can be more consistent with the reality. At the same time, a thin film with a high defect density and capture cross section was inserted between the perovskite films and HTL to characterize interface deterioration, improving the reliability of the simulation. In our design, this new method of combining experiment and simulation was used to explore device property. So far, almost no reports have been found that compare the performance of different hole transport layers through the combination of experiment and simulation as we have. This method is of great significance for research into PSCs.

## 2. Experimental and Simulation

### 2.1. Materials

Fluorine-doped SnO_2_ (FTO), nickel oxide solution (NiO_x_), and isopropanol (IPA) were purchased from Shanghai MaterWin New Materials Co., Ltd. (Shanghai, China). Dimethyl sulfoxide (DMSO) and N,N-dimethyl formamide (DMF) were purchased from Alfa Aesar (China) Co., Ltd. (Shanghai, China). Poly(3,4-ethylenedioxythiophene): poly (styrenesulfonate) (PEDOT:PSS), methylammonium iodide (MAI), and PbI_2_ were obtained from Xi’an Polymer Light Technology Corp (Xi’an China).

### 2.2. Device Fabrication

The FTO substrates were prepared by ultrasonic cleaning with the alcohol, glass water (2-propanol:deionized water:acetone = 1:1:1), and the blended solution (deionized water and detergent) for 15 min, respectively. The substrates were cleaned by ultraviolet ozone (UVO) for 90 min before spin-coating. The PEDOT:PSS solution was diluted in IPA, and then deposited on bottom substrate for 20 s at 1000 rpm. The substrate of FTO/PEDOT:PSS was placed on a hot plate for 5 min at 120 °C. Nickel oxide solution was deposited on the bottom substrate for 30 s at 4000 rpm. The FTO/NiO_x_ substrate was annealed on a heating plate for 10 min at 150 °C and then calcined for 1 h at 350 °C in a muffle furnace with ceramic fiber (Huagang Tong Technology, Beijing, China). The PbI_2_ powder, completely dissolved in mixed solution of DMF and DMSO (volume ration = 1:4), spin-coated on FTO/PEDOT:PSS or FTO/NiO_x_ substrate for 30 s at 1500 rpm. Furthermore, the MAI powder was dissolved in isopropanol solution and deposited on the PbI_2_ layer for 30 s at 1500 rpm. Finally, the samples were placed on a heating plate for 15 min at 150 °C.

### 2.3. Characterization

The microstructure of perovskite films was characterized by scanning electron microscopy (SEM) (Sigma, Zeiss, Jena, Germany). The absorption spectra of perovskite layers onto different substrates were analyzed by an ultraviolet (UV) visible absorption spectrometer (Avantes, Apeldoom, The Netherlands), and the photoluminescence spectra were examined by a LabRAW HR800 PL testing system (HORIBA Jobin Yvon, Paris, France).

### 2.4. Device Simulation Parameters

**Figure 1:**
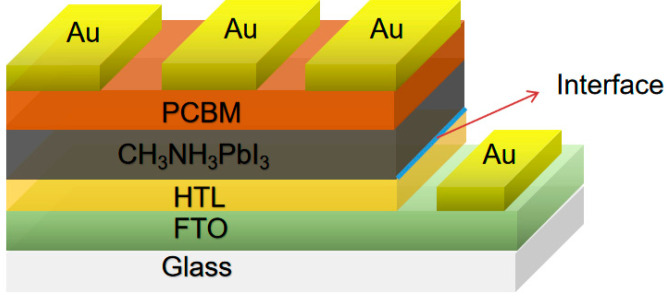
The device structure for simulation design.

The device was designed with inverted structure by using sing wxAMPS. The temperature of the simulation was 300 K. The conventional parameters are listed in Table 1, as described in the results and discussion. The functional settings were as follows: defect type was neutral and the center of bandgap (*E_g_*) was selected as defect energy level. The original defect densities of HTL and perovskite layer were 10^13^ cm^−3^ and 10^14^ cm^−3^, respectively. Gaussian energetic distribution was selected, and its characteristic energy was 0.1 eV. The original capture cross-section of function layers were 10^−14^ cm^2^. The work function of bottom and left electrode are 5.1 eV (Au) and 4.4 eV (FTO), respectively. The condition of AM1.5 illumination was applied to all simulations. A thin film (10 nm) was inserted between HTL and perovskite layer and its parameters were selected from the light absorption layer. Figure 1 shows the constitution of the PSCs. The electron transport layer we use in the simulation is [6,6]-phenyl-C61-butyric acid methyl ester (PCBM).

## 3. Results and Discussion

PEDOT:PSS and NiO_x_ were adopted as organic and inorganic HTLs for inverted PSCs, respectively. Since the position of the valence band of NiO_x_ is closer to that of CH_3_NH_3_PbI_3_ (MAPbI_3_), the open-circuit voltage (V_oc_) of the PSCs with NiO_x_ as the HTL is higher than PEDOT:PSS [39]. The deposition of perovskite films on different organic substrates was studied in previous reports [21]. It was observed that different bottom substrates would had an influence on the growth of perovskite crystals.

**Figure 2:**
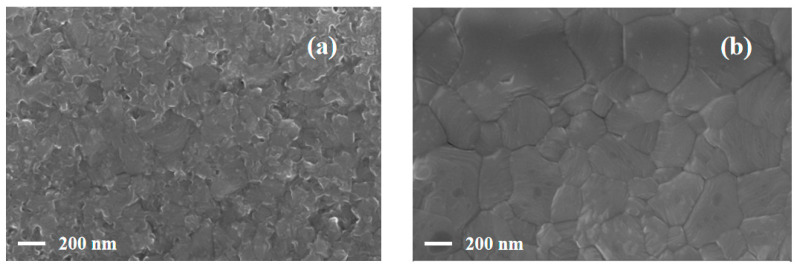
Scanning electron microscope (SEM) top-view of perovskite layers: (**a**) the sample of fluorine-doped SnO_2_/polyethylene dioxythiophene:poly (styrene sulfonate) (FTO/PEDOT:PSS/MAPbI_3_); (**b**) the sample of FTO/NiO_x_/MAPbI_3_.

The perovskite layer deposited on NiO_x_ has a low defect density and a large grain size according to the literature report [39]. Figure 2 displays the surface morphology of perovskite layers grown on two kinds of bottom layer. The grain size of perovskite layer is smaller in Figure 2a and the accumulation of grains leads to the decrease in film flatness, which may result in the high defect density. From Figure 2b, the integral grains with large size and obvious grain boundaries are obtained. There are no pinholes on the surface and the results indicate that the NiO_x_ bottom layer is more conducive to the deposition of the perovskite layer. The low-quality surface morphology may be ascribed to the deterioration of the interfacial layer.

**Figure 3:**
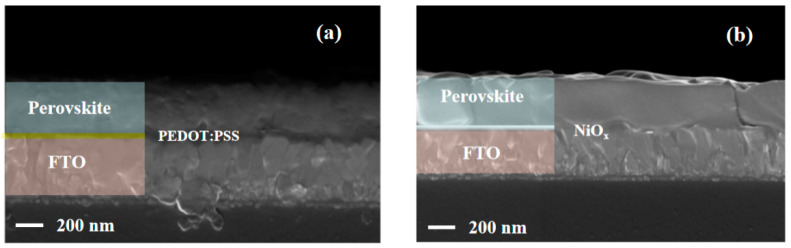
Cross-sectional SEM images: (**a**) the sample of FTO/PEDOT:PSS/MAPbI3; (**b**) the sample of FTO/NiO_x_/MAPbI3.

To make out the influence of different HTLs on the morphology and photophysical characteristic of perovskite layers, two kinds of perovskite film with different structures (FTO/PEDOT:PSS/MAPBI_3_ and FTO/NiO_x_/MAPBI_3_) were prepared. From Figure 3a, the perovskite film deposited on the bottom layer has poor flatness. Past research has revealed that the hydrophilicity of PEDOT:PSS depends on the polar solvents treatment, which may impact the grain size and morphology of the perovskite [40,41]. In Figure 3b, NiO_x_ is selected as the bottom layer of perovskite film. Compared to Figure 3a, the interface between the HTL and perovskite film is flatter. In addition, the perovskite film is compact with high quality. The main reason that PEDOT:PSS shows hygroscopicity is due to the nature of PSS (polystyrene sulfonate) [42]. Oxygen and humidity in PEDOT:PSS diffuse into the perovskite layer through the interface [43]. Perovskite structure would hydrolyze and degrade in a humid environment due to its sensitivity to moisture and oxygen, resulting in the destruction of the layered structure [26,27]. According to the report, conductive glass substrates could also be corroded by acidity, causing their components to invade the organic layer [30].

**Figure 4:**
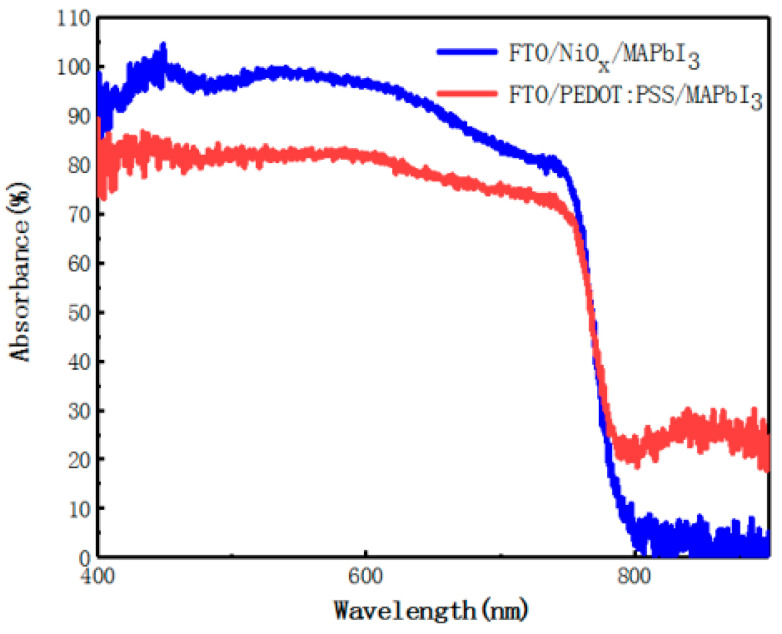
Ultraviolet–visible (UV–Vis) absorption spectra of perovskite films on different hole transport layers (HTL).

To research the impact of perovskite growth on different HTLs, the UV–vis absorption of light-absorption layers grown on two kinds of substrates (PEDOT:PSS and NiO_x_) are compared (Figure 4). Although there is no change in the composition of the perovskite, the enhancement of light absorption of the perovskite film deposited on NiO_x_ layer can be seen in the wavelength ranging from 400 nm to 760 nm, which is due to improved surface morphology of perovskite film and higher optical transmittance of the NiO_x_ layer.

**Figure 5:**
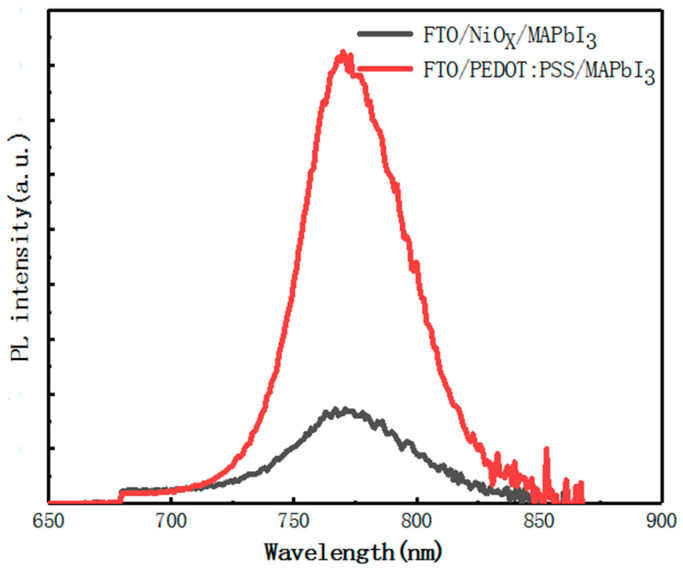
Photoluminescence spectra of perovskite light-absorption layer films deposited on different HTLs.

The photoluminescence spectra of ITO/HTLs/perovskite structure are obtained, as shown in Figure 5. Both of the photoluminescence emission peaks are located at about 765 nm. However, the emission peaks of perovskite layers grown on organic HTL (PEDOT:PSS) was significantly increased, which was attributed to the weak hole transport capacity of PEDOT: PSS compared with that of NiO_x_. When the holes are transported away, the recombination of carriers is reduced, resulting in a decrease in photoluminescence intensity. Generally speaking, the lower the photoluminescence intensity, the better the device performance.

To improve the accuracy of simulation results, we set the perovskite layers deposited on the two hole transport layers with different optical absorption coefficients in the software for simulation, the parameters of which come from the absorption spectra (Figure 4). Furthermore, the above experimental data and the physical parameters of the material are combined to simulate for PSCs device by using wxAMPS. The simulation design will be introduced in the following content.

**Figure 6:**
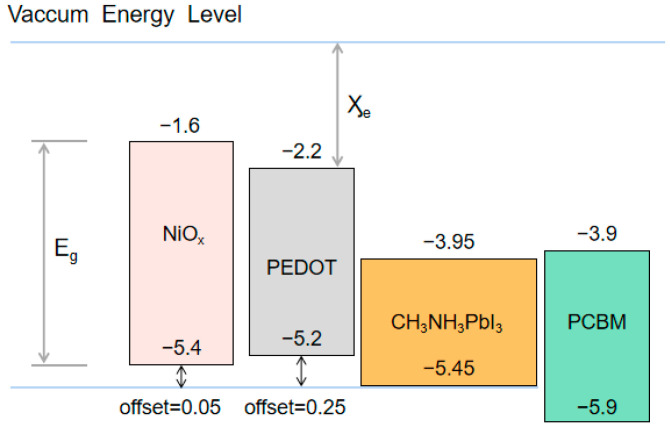
Energy diagrams of the constituting layers with different HTL materials.

The physical parameters of different functional layers are provided in Table 1. E_g_ represents the band gap of different materials; Ӽ_e_ is the electron affinity; ε_r_ is the dielectric constant; μ_n_ and μ_p_ are the electron and hole mobility of the functional layer, respectively; NA and ND are the acceptor and donor impurity concentration; NC and NV are the effective density of states (DOS) of the valence band and conduction band, respectively; Value band offset is defined as the difference between the maximum value band (MVB) of the light absorbing layer and the hole transport layer. Energy diagrams of the constituting layers with different HTL materials are shown in Figure 6. These parameters are adopted from references [1,2,3,4,5,6,7,8].

The choice of hole transport layer is very important in inverted PSCs. Since light is incident from the hole transport layer side, the optical transmittance and absorption coefficient of the layer should also be considered. The introduction of these parameters will make the simulation results more accurate. As an inorganic material, NiO_x_ has good optical transmittance. To better simulate the impact of the NiO_x_ film on a device, the absorption coefficient of the material is calculated and is written as:

(1) $$K=\frac{\alpha \lambda }{\pi }$$

where *K* is the extinction coefficient, *α* is the absorption coefficient, and *λ* is the wavelength. The parameters are obtained from the reports [44,45]. The absorption coefficient of the PEDOT:PSS layer is given by the Lambert–Beer law from:

(2) $$A(\lambda )=\mathrm{lg}(\frac{1}{T})=\alpha (\lambda )\;\times \;l\;\times \;c$$

where *A*(*λ*) is the absorbance; *T* is the light transmittance; *l* is the thickness of the absorbing layer and *c* is the concentration of the substance [46].

**Figure 7:**
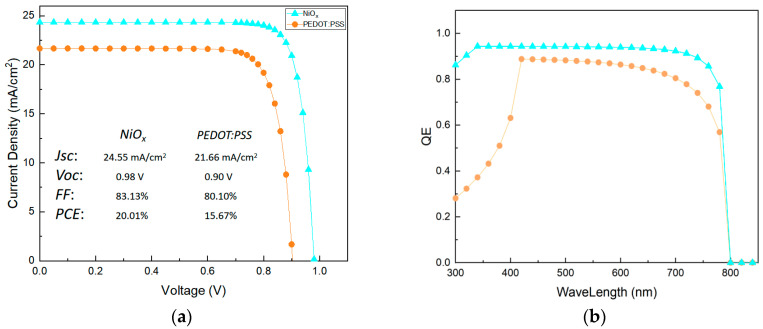
Simulation results of the proposed PSC with different HTL (**a**) J-V curves (**b**) quantum efficiency (QE) curves.

Figure 7a shows the J-V (current density-voltage) curves of the design PSCs with different HTLs. We can observe that the short-circuit current density (*J_sc_*) of the PSCs with PEDOT as the HTL has a significant reduction due to the change of photophysical property of the perovskite film. The open-circuit voltage loss is also greater than that of device with NiO_x_ as the HTL. The perovskite film deposited on NiO_x_ layer has a high-quality morphology, so its defect density is low, resulting in a reduction in non-radiative recombination. The device series resistance increase is caused by the low hole mobility of the organic HTL and some point defects of the perovskite layer, which results in reduction of the fill factor. Therefore, the selection of the hole transport layer has an impact on the performance of the PSCs. After many simulations, it was found that when the thickness of perovskite on NiO_x_ substrate is 390 nm, the power conversion efficiency (PCE) has the best value of 20.09%. When the thickness of perovskite on the PEDOT substrate is 620 nm, the best power conversion efficiency is 15.85%. The PCEs and J-V curves simulation results are as shown in Tables S1 and S2, Figures S1 and S2 (Supplementary Materials).

The quantum efficiency (QE) of devices with different HTLs is shown in Figure 7b. We can observe that the quantum efficiency of the device with NiO_x_ as the HTL rises rapidly to 0.95, in the wavelength range of 300–340 nm, reaching the maximum. The difference in quantum efficiency in the front stage is caused by the difference in optical transmittance of the hole transport layer, which is reflected in the optical absorption coefficient and film thickness of the HTL. The device with NiO_x_ as the HTL maintains a s quantum efficiency in the wavelength range of 340–750 nm, maintaining above 90%. However, the quantum efficiency of the device with PEDOT as the HTL shows significant decline within the same range, and the value is lower than that of the NiO_x_ device. This is due to the poor morphology, and film thickness of the perovskite grown on different HTL materials. The difference is reflected in the setting of the optical absorption coefficient of the perovskite layers of different devices (refer to the absorption spectra). In addition, the decrease of quantum efficiency may be due to the lower electron and hole mobility of the PEDOT:PSS layer, which affects the collection of charge.

As mentioned before, the HTL is used as bottom side for the inverted PSCs, which impacts the growth of perovskite film. Research shows in the reference that the defect density of the interlayer between the perovskite film and the HTL has a significant influence on the performance of the device. It can be made out that the perovskite film grown on organic HTL has a structural deterioration at the interlayer from the SEM images. PEDOT:PSS showed hygroscopic due to the nature of PSS [42]. Moisture and oxygen will diffuse into the light absorption layer through the HTL, causing decomposition of perovskite and a lot of point defects at the interface [43]. On the original structure of PSCs, it is difficult to quantify the interface defect density. Therefore, an interfacial layer is inserted between HTL and perovskite film. By selecting parameters from the perovskite layer, the thickness of the film is determined to be 10 nm. However, the interface layer is of high defect density and high capture cross-section because of the destruction of the structure.

**Figure 8:**
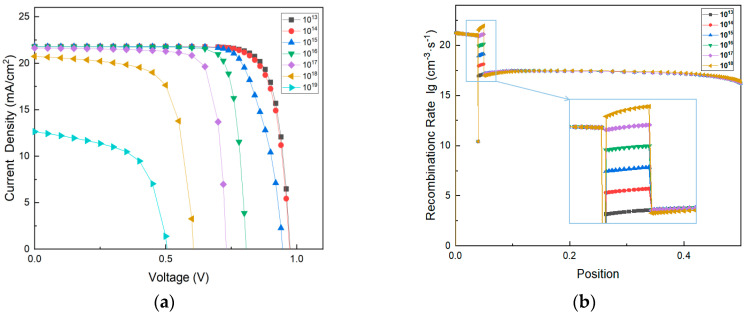
Simulation results of the proposed PSC with different defect density (cm^−3^) of the interface between PEDOT:PSS and CH_3_NH_3_PbI_3_ (**a**) J-V curves (**b**) recombination rate.

The J-V curves of devices with various defect densities of interface layer are shown in Figure 8a. It can be observed that the *V_oc_* of the device decreases unlinearly with the increase of defect density of the interface layer. The *V_oc_* of the PSCs is the same as the case of the original device, as the defect density is 10^14^ cm^−3^. As the defect density increases, the loss of *V_oc_* enhances significantly. In addition, the presence of the defect states causes a slow reduction in *J_sc_* in the order of 10^13^ cm^−3^–10^18^ cm^−3^ and then dropping to about 13 mA/cm^2^ when the defect density is 10^19^ cm^−3^.

Due to the increase of the non-radiative recombination at the interface, the deep trap energy levels generated by several point defects result in the decrease of the device performance. The recombination rate of devices with different interface defect density is shown in Figure 8b. We can find that as the defect density increases, the recombination rate of the device enhances significantly at the position of 40–50 nm, which corresponds to the interlayer with a thickness of 10 nm. The point defects of deteriorated interface cause the generation of deep trap energy levels, which leads to the trap-assisted non-radiative recombination. The defect density magnitude was set in the simulation software varies from 10^14^ cm^−3^ to 10^19^ cm^−3^, which greatly affects the charge transport and recombination rate of devices. When the defect density is high, the decrease in *J_sc_* and a significant voltage loss can be observed. Therefore, the increase in defect density generates a negative influence on device performance.

**Figure 9:**
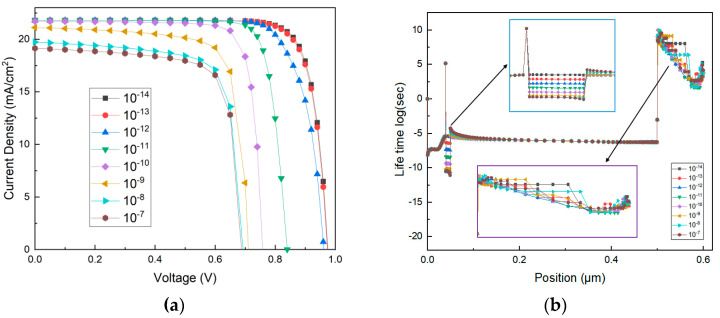
Simulation of the proposed PSC with capture cross-section (cm^2^) of interface between PEDOT:PSS and CH_3_NH_3_PbI_3_ (**a**) J-V curves (**b**) hole lifetime.

The initial capture cross-section of electrons and holes is 10^−^^14^ cm^2^, which gives a carrier diffusion length of about 100 nm [47]. To illustrate the deterioration mechanism of the heterojunction, a thin functional layer with high capture cross-section is inserted between perovskite layer and PEDOT:PSS to research the effect of carrier lifetime on device property. The J-V curves with various capture cross sections of interface layers are shown in Figure 9a. The enhancement of capture cross section results in the voltage loss and decrease in *J_sc_* in the range of 10^−^^14^ cm to 10^−^^7^ cm. This indicates that the high capture cross section of the interface layer generates a negative influence on the device performance.

The degradation of device performance may be due to the enhancement of the recombination rate and reduction in carrier lifetime caused by the high capture cross-section at the interface. To account for the phenomenon, the interface layer is considered with capture cross-section varying from 10^−14^ cm^2^ to 10^−7^ cm^2^. As shown in Figure 9b, the reduction of the hole lifetime is observed in the range of 40–50 nm, corresponding to the position of the interface layer. Lifetime and recombination will affect the carrier diffusion length, which has a marked affect on the device performance. It is worth noting that the enhanced capture cross-section of the interlayer affects the carrier lifetime of electron transport layer.

## 4. Conclusions

In this study, the morphology and optical absorption of perovskite layers grown on different HTLs were compared. The spin-coating on different hole transport layers will affect the morphology of perovskite. Therefore, there is a certain relationship between the absorption coefficient and the morphology when the simulation is carried out in this case. The NiO_x_ bottom layer is conducive to the growth of flat perovskite with high-quality morphology and enhancement of absorption. Then, the optical absorption parameters were introduced to the simulation software for the device design. To clarify the deteriorating interface between perovskite layer and PEDOT: PSS, a thin film with high defect density and capture cross-section was inserted, which caused the increase in recombination and reduction of carrier lifetime. The introduction of interface layer had a negative impact on device performance, manifested in the voltage loss and decrease in current density. The device design established in our work has performed well in both experiment and simulation, which supported the investigation of the impact of HTL materials and parameters of interlayer on device performance. The method combining experimental and simulated approaches, rarely used in previous studies, has great potential in studying the properties of PSCs.

## Figures and Tables

**Table 1:** Basic parameters for simulation of perovskite solar cells (PSCs) in this study [1,2,3,4,5,6,7,8].

Basic Parameters	NiO_x_	PEDOT:PSS	Interface Layer	CH_3_NH_3_PbI_3_	PCBM
E_g_ (eV)	3.8	3	1.5	1.5	2
Ӽe (eV)	1.6	2.2	3.95	3.95	3.9
ε_r_	11	7.5	30	30	3.9
Thickness (nm)	30	40	10	500/450	100
μ_n_ (cm^2^V^−1^s^−1^)	2.8	0	14	14	0.2
μ_p_ (cm^2^V^−1^s^−1^)	2.8	0.001	14	14	0
N_A_(cm^−^^3^)	1.5 × 10^18^	1 × 10^18^	6 × 10^14^	6 × 10^14^	0
N_D_(cm^−3^)	0	0	0	0	2.93 × 10^17^
N_C_(cm^−3^)	1 × 10^18^	1 × 10^16^	2.5 × 10^20^	2.5 × 10^20^	2.5 × 10^21^
N_V_(cm^−3^)	1 × 10^18^	1 × 10^19^	2.5 × 10^20^	2.5 × 10^20^	2.5 × 10^21^
Value band offset (eV)	0.05	0.25	-	-	-

## Data Availability

The data used to support the findings of this study are available from the corresponding author upon request.

## Supplementary Materials

The following are available online at https://www.mdpi.com/article/10.3390/ma14092191/s1, Table S1: Device efficiencies for different perovskite film thicknesses on NiO_x_, Table S2: Device efficiencies for different perovskite film thicknesses on PEDOT:PSS, Figure S1: J-V curves of the device efficiencies for different perovskite film thicknesses on NiO_x_, Figure S2: J-V curves of the device efficiencies for different perovskite film thicknesses on PEDOT:PSS.
