# Supplementary material for: Device Modeling and Design of Inverted Solar Cell Based on Comparative Experimental Analysis between Effect of Organic and Inorganic Hole Transport Layer on Morphology and Photo-Physical Property of Perovskite Thin Film
Source: Materials (Basel). 2021 Apr 24;14(9):2191. doi: 10.3390/ma14092191 (PMC8123108; doi:10.3390/ma14092191)
Supplement: Supplementary file 1 [file materials-14-02191-s001.zip › materials-1132050-supplementary.pdf]

materials MDPI

Supplementary Materials

D Device Modeling and Design of Inverted Solar Cell Based on Comparative Experimental Analysis Between Effect of Organic and Inorganic Hole Transport Layer on Morphology and Photo-Physical Property of Perovskite Thin Film

Xiaolan Wang, Xiaoping Zou *, Jialin Zhu *, Chunqian Zhang, Jin Cheng, Zixiao Zhou, Haiyan Ren, Yifei Wang, Xiaotong Li, Baokai Ren and Keke Song

Beijing Advanced Innovation Center for Materials Genome Engineering, Research Center for Sensor Technology, Beijing Key Laboratory for Sensor, MOE Key Laboratory for Modern Measurement and Control Technology, School of Automation, Beijing Information Science and Technology University, Jianxiangqiao Campus, Beijing 100101, China; wangxl1105@163.com (X.W.);chun-qiancool@163.com (C.Z.); chengjin@bistu.edu.cn (J.C.); 18049217206@163.com (Z.Z.); yanh3100@gmail.com (H.R.); yifewang2020@126.com (Y.W.); xiaotong252240@163.com (X.L.);renbk2021@163.com (B.R.); song-mengke163@163.com (K.S.)

* Correspondence: xpzou2014@163.com (X.Z.); jlzhu@bistu.edu.cn (J.Z.); Tel.: +86-1364-105-6404 (X.Z.)

Table S1. Device efficiencies for different perovskite film thicknesses on NiO_x.

Thickness (nm)	PCE (%)
350	20.0698
360	420.0806
370	20.0875
380	20.0903
390	20.0904
400	20.0875
450	20.0671
500	20.0101
550	19.9525

Table S2. Device efficiencies for different perovskite film thicknesses on PEDOT:PSS.

Thickness (nm)	PCE (%)
400	15.5219
450	15.6779
500	15.7752
550	15.8231
600	15.8532
610	15.8553
620	15.8561
630	15.8558

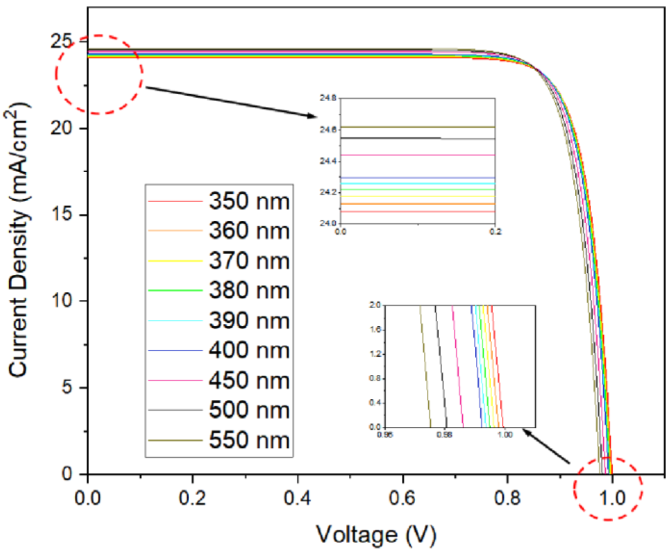

Figure S1. J-V curves of the device efficiencies for different perovskite film thicknesses on NiO_x.

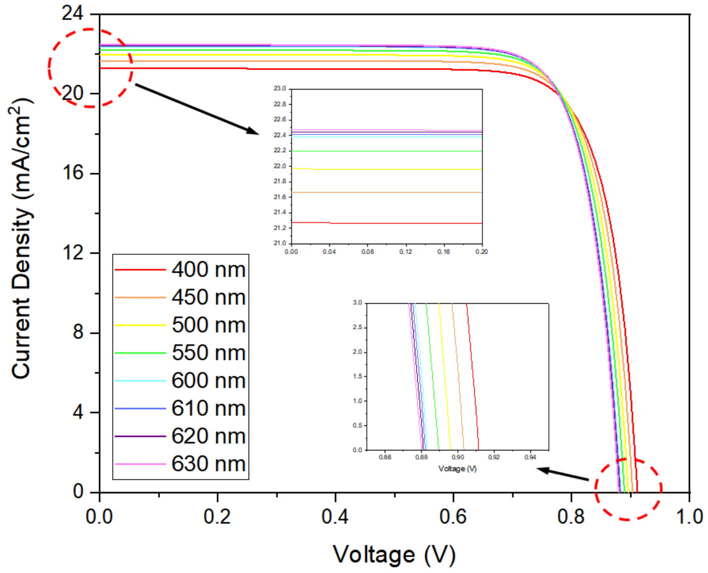

Figure S2. J-V curves of the device efficiencies for different perovskite film thicknesses on PE-DOT:PSS.
